# Rhinovirus Load Is High despite Preserved Interferon-β Response in Cystic Fibrosis Bronchial Epithelial Cells

**DOI:** 10.1371/journal.pone.0143129

**Published:** 2015-11-23

**Authors:** Nurlan Dauletbaev, Mithun Das, Maria Cammisano, He Chen, Sareen Singh, Cora Kooi, Richard Leigh, Trevor Beaudoin, Simon Rousseau, Larry C. Lands

**Affiliations:** 1 Research Institute of McGill University Health Centre, Montreal, Quebec, Canada; 2 Department of Medicine and Physiology and Pharmacology, University of Calgary, Calgary, Alberta, Canada; 3 Meakins-Christie Laboratories, McGill University, Montreal, Quebec, Canada; 4 Respiratory Division, Montreal Children’s Hospital, Montreal, Quebec, Canada; University of Alabama at Birmingham, UNITED STATES

## Abstract

Lung disease in cystic fibrosis (CF) is often exacerbated following acute upper respiratory tract infections caused by the human rhinovirus (HRV). Pathophysiology of these exacerbations is presently unclear and may involve deficient innate antiviral or exaggerated inflammatory responses in CF airway epithelial cells. Furthermore, responses of CF cells to HRV may be adversely affected by pre-exposure to virulence factors of *Pseudomonas* (*P*.) *aeruginosa*, the microorganism that frequently colonizes CF airways. Here we examined production of antiviral cytokine interferon-β and inflammatory chemokine interleukin-8, expression of the interferon-responsive antiviral gene 2’-5’-oligoadenylate synthetase 1 (*OAS1*), and intracellular virus RNA load in primary CF (delF508 *CFTR*) and healthy airway epithelial cells following inoculation with HRV16. Parallel cells were exposed to virulence factors of *P*. *aeruginosa* prior to and during HRV16 inoculation. CF cells exhibited production of interferon-β and interleukin-8, and expression of *OAS1* at levels comparable to those in healthy cells, yet significantly higher HRV16 RNA load during early hours post-inoculation with HRV16. In line with this, HRV16 RNA load was higher in the CFBE41o- dF cell line overexpessing delF508 *CFTR*, compared with the isogenic control CFBE41o- WT (wild-type *CFTR*). Pre-exposure to virulence factors of *P*. *aeruginosa* did not affect *OAS1* expression or HRV16 RNA load, but potentiated interleukin-8 production. In conclusion, CF cells demonstrate elevated HRV RNA load despite preserved interferon-β and *OAS1* responses. High HRV load in CF airway epithelial cells appears to be due to deficiencies manifesting early during HRV infection, and may not be related to interferon-β.

## Introduction

Cystic fibrosis (CF) is a genetic multiorgan disease, in which infectious and inflammatory processes in the airways (CF lung disease) largely determine morbidity and premature demise. CF lung disease is often exacerbated following acute upper respiratory tract infections (i.e., the “common cold”) [1, 2], which are most commonly caused by human rhinoviruses (HRV) [1–4]. Virus-associated exacerbations are burdensome for patients and the health care system. The mechanisms of these exacerbations in CF are presently unclear. To elucidate these, several studies have been carried out to date, but the obtained information is still fragmentary, necessitating further investigation on this topic.

HRV are positive-strand RNA viruses that replicate in airway epithelium of both upper and lower airways, causing innate antiviral responses. Central to these responses is production of interferon (IFN) -β, which up-regulates expression of interferon-responsive genes, such as 2’-5’-oligoadenylate synthetase 1 (*OAS1*), that degrade viral nucleic acids, block translation of viral proteins, and attract immune cells to the airways. Antiviral immunity limits HRV infections to the upper airways of healthy individuals, but seems to be less efficient in CF, allowing the infection to spread to the lower airways. This was demonstrated in exacerbated patients with CF whose lower airway secretions yielded high HRV load [5].

In addition to potentially abnormal antiviral responses, infected CF airway epithelial cells may produce excessive amounts of inflammatory factors, such as interleukin (IL) -8, the major neutrophil chemoattractant. CF airways are chronically inflamed, and the levels of IL-8 increase further during virus exacerbations [5].

In addition, innate antiviral and inflammatory responses in CF airways may be altered by constant exposure to bacterial virulence factors. CF airways harbour a panel of opportunistic bacteria, with *Pseudomonas (P*.*) aeruginosa* being the most prominent microorganism. Airway epithelium comes in direct contact with *P*. *aeruginosa* during initial steps of infection, but further direct contact may be less frequent following formation of bacterial biofilm situated in the airway lumen [6]. Nonetheless, CF airway epithelial cells are likely to be constantly exposed to virulence factors that leach out of the biofilm [7].

Here we sought to assess IFN-β and IL-8 responses, expression of the interferon-responsive antiviral gene *OAS1*, and intracellular virus RNA load, in primary CF and healthy airway epithelial cells infected with HRV. In addition, we tested potential adverse effects of bacterial virulence factors on HRV-stimulated IFN-β, IL-8, and *OAS1* responses, and HRV RNA load.

## Materials and Methods

### Reagents and assays

Cell culture flasks and plates were from Fisher Scientific (Ottawa, Canada). Supplies for culture of primary bronchial epithelial cells were from Lonza (Walkersville, USA). Minimal Essential Medium (MEM) was purchased from Life Technologies (Burlington, Canada), while Foetal Bovine Serum (FBS) was obtained from Wisent (Saint-Jean-Baptiste, Canada). Coating solution to promote cell attachment to culture vessels was from Advanced Biomatrix (bovine collagen solution type I; San Diego, USA) or made per our previous protocol [8, 9]. Verikine IFN-β high-sensitivity serum ELISA kit and IL-8 ELISA set were respectively from PBL Assay Science (Piscataway, USA) and BD Biosciences (Mississauga, Canada). Supplies for RNA isolation (RNeasy micro kit), reverse transcription (Quantitect RT kit), and qPCR (primers and Quantifast Sybr Green qPCR kit) were purchased from Qiagen (Toronto, Canada). The HRV16 virus RNA quantification kit was from Primer Design (Southampton, UK). The ViewRNA *in situ* hybridization kit, HRV positive and negative strand probes, and *ACTB* probe were from Affymetrix (Santa Clara, USA). *P*. *aeruginosa* flagellin and *Escherichia* (*E*.) *coli* lipopolysaccharide (LPS) were purchased from Invivogen (San Diego, USA). Recombinant human IL-1β was obtained from BD Biosciences. The 12-mm, #1 glass coverslips were from NeuVitro (Vancouver, USA), whereas glass slides were purchased from Fisher Scientific. The electron microscopy grade, 16% paraformaldehyde was from Electron Microscopy Sciences (Hatflield, USA), and Prolong Diamond fluorescence mounting medium was from Life Technologies. All other supplies were from Sigma-Aldrich (Mississauga, Canada).

### Primary cells

For principal experiments, we used primary bronchial epithelial (HBE) cultures from eight healthy and eight CF individuals provided by the Primary Cell Airway Biobank at McGill Cystic Fibrosis Translational Research Centre (CFTRc; Montreal, Canada). The use of CF or healthy donor lungs to obtain primary airway epithelium was approved by the Human Ethics Board of Centre hospitalier de l’Université de Montréal. Written informed consents were obtained from the donors. Consent forms were approved by the Institutional Review Board of the Research Ethics Office of McGill University. Healthy HBE cells were procured from discarded lungs of transplant donors with no known lung disease and were confirmed to express wild-type CFTR. CF cells were obtained from CF lungs removed at lung transplantation. CF donors included 7 male and 1 female adult patients whose ages ranged from 19 to 41 years. Five CF donors were homozygous for the delF508 *CFTR* mutation (the most common mutation among Caucasian patients), while the remaining three were from patients heterozygous for the above mutation, with the other allele respectively being 711+1G>T, 621+1G>7, and an unknown mutation. Both healthy and CF HBE cells were propagated and frozen at passage 1.

Two healthy HBE cultures were purchased from Lonza and used in preliminary experiments.

**Fig 1:**
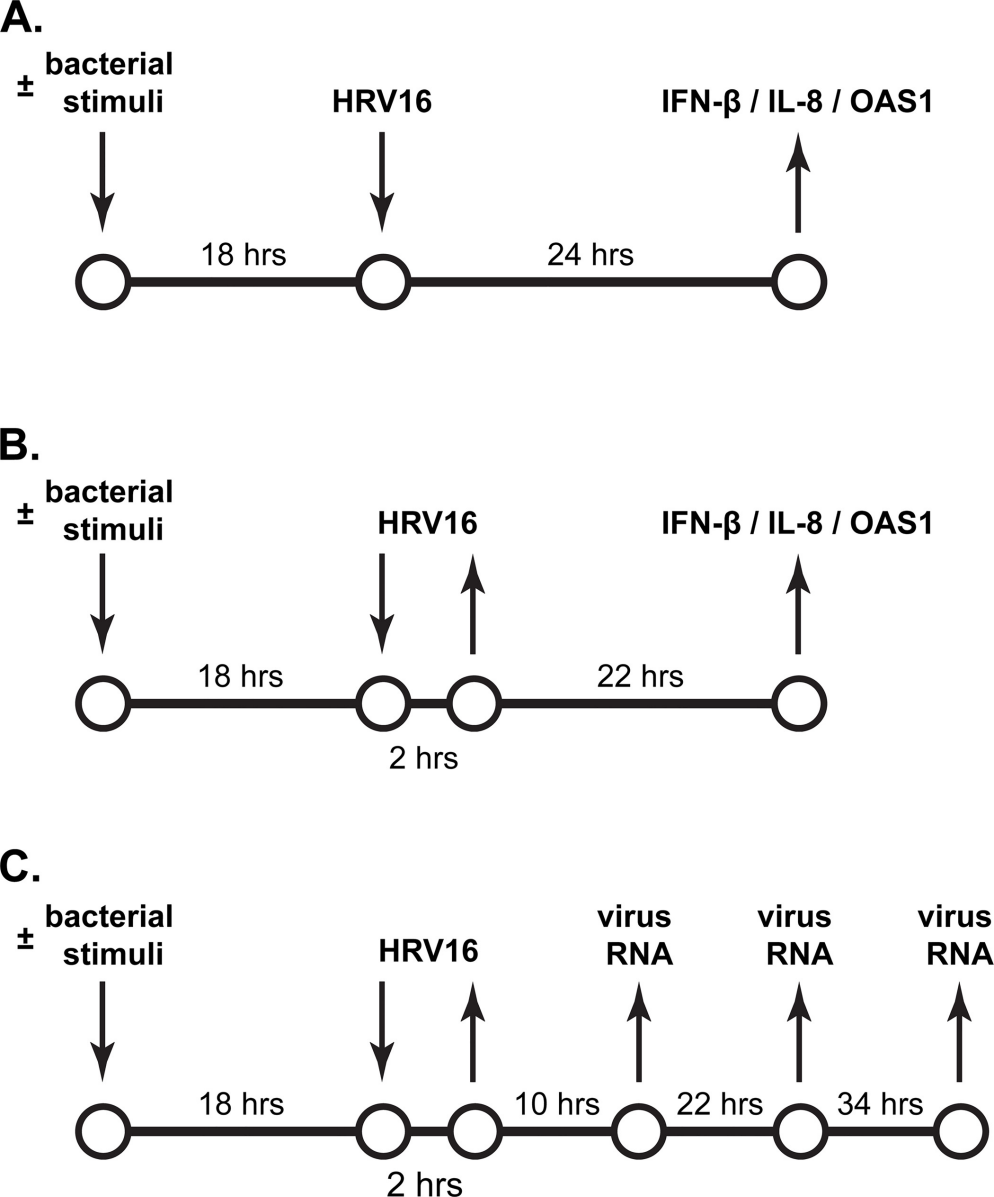
Schematic representation of continuous and short-term inoculation with HRV, and the tested outcomes. (**A**) Continuous inoculation with HRV16. Cells were incubated with culture medium for 18 hours and subsequently inoculated for 24 hours with the human rhinovirus (HRV) 16 at a Multiplicity Of Infection (MOI) of 0.1. Afterwards, IFN-β and IL-8 production was quantified in cell supernatants by ELISA, whereas expression of IFN-β and IL-8 mRNAs, and the interferon-responsive antiviral gene *OAS1* was assessed in cell lysates by qPCR. Parallel cells were incubated with diffusible virulence factors of *P*. *aeruginosa* (“bacterial stimuli”) prior to and during inoculation with HRV16. (**B**) Short-term inoculation with HRV16. Cells were treated as in (**A**) prior to inoculation for 2 hours with HRV16 at an MOI of 0.1. Then, cell supernatants were removed, and cells were rinsed twice with culture medium to deplete extracellular virus. Subsequently, the cells were incubated for 22 hours without HRV16, and with or without virulence factors of *P*. *aeruginosa*. Afterwards, IFN-β and IL-8 (protein and mRNA) response, and expression of the interferon-responsive antiviral gene *OAS1* were quantified as in (**A**). (**C**) Quantification of intracellular HRV RNA load after a short-term inoculation with HRV16. Primary healthy and CF HBE cells were treated and inoculated as in (**B**). Then, cells were incubated without HRV16, and with or without diffusible virulence factors of *P*. *aeruginosa*. Cell lysates were collected at 10, 22, and 34 hours post-inoculation with the virus. HRV16 RNA copy numbers were quantified by qPCR.

For experiments, frozen cells (passage 1) were thawed, counted, plated onto coated 24-well culture plates at a concentration of 10,000/cm^2^, and grown under submerged conditions in BEGM culture medium supplemented with growth factors, retinoic acid, hydrocortisone, and antibiotics. At 70–80% confluency, culture medium was changed to experimental BEGM that contained growth factors and retinoic acid, but no hydrocortisone or antibiotics (Fig 1). Both latter medium constituents were removed to avoid potential alteration of cell IFN-β or IL-8 responses. In some experiments, bacterial virulence factors (described below and in the Results section) were added to HBE cells along with experimental BEGM (Fig 1). After pre-incubation for 18 hours, the cells were infected with HRV16 in experimental culture medium, with or without ongoing exposure to bacterial virulence factors (Fig 1). Afterwards, cell supernatants were collected for IFN-β or IL-8 ELISAs, while cells were lysed in 80 μl of Buffer RLT (RNeasy micro kit). Both supernatants and lysates were stored at -80°C pending analyses.

Each culture represented an individual observation point, such that “n = 8” meant experiments conducted with cells from 8 different donors. Most experiments were done using passage 1 cells. Some experiments were conducted with passage 2 cells, if the recovery of freshly thawed cells was low. Passage 2 cells were obtained by propagating cells from frozen stocks in coated T75 flasks. If different passage cells were obtained from the same donor, these were never used in the same experiment to preserve the inter-individual variability of cell responses.

### CFBE41o- parental and stably transformed cell lines

In some experiments, we used an immortalized airway epithelial cell line CFBE41o-. This cell line is homozygous for delF508 *CFTR* and is henceforth referred to as “parental CFBE41o-”. Besides parental CFBE41o-, we utilized two stably transformed cell lines derived from it: CFBE41o- WT and CFBE41o- dF. These transformed cell lines are isogenic cell lines that respectively overexpresses wild-type or delF508 *CFTR*. All three cell lines were a kind gift of Dr. Dieter Gruenert (UCSF, San Francisco, USA) and have been described in details elsewhere [10, 11].

Parental CFBE41o- cells were cultured in MEM as previously done by us [8, 9]. Transformed CFBE41o- WT and dF cell lines were cultured in MEM supplemented with 10% FBS, 200 mM L-glutamine. The medium was further supplemented with Hygromycin B (Life Technologies) to maintain overexpression of *CFTR*. For experiments, parental and transformed CFBE41o- were counted, plated onto coated 24-well plates at a concentration of 0.25 × 10^6^ cells/ml, and grown for 18 hours. During these 18 hours, transformed cell lines were deprived of Hygromycin B to minimize confounding effects of this selection antibiotic on HRV infection. Then, cells were infected with HRV16 in antibiotic-free MEM supplemented with 2% FBS and 200 mM L-glutamine. After infection, cell supernatants and lysates were collected for various outcomes similar to primary HBE cells.

### HRV propagation and infection

We utilized HRV16 which is a genotype A and major group HRV strain that binds to airway epithelium via ICAM-1. This strain is frequently used in the literature to study innate antiviral immunity in cells from patients with chronic inflammatory disease, including CF. The virus was propagated and purified as previously described [12] and utilized at a 50% Tissue Culture Infective Dose (TCID_50_) of 10^5.85^/ml. To infect primary healthy and CF HBE cells and immortalized cell lines, virus stock was added to cell supernatants to yield a desired Multiplicity Of Infection (MOI; in the majority of experiments, MOI of 0.1). To determine the virus-to-cell ratio and calculate the MOI, cells on parallel wells were lifted and counted.

HRV infect a small percentage of airway epithelial cells and do not cause extensive cytotoxicity [13–16]. The previously published studies of HRV infection in CF airway epithelial cells mostly utilized high inoculation doses of the virus (MOI of ≥1) which (i) may lead to cytotoxicity, (ii) may increase the percentage of infected cells and/or (iii) increase the likelihood that the same cell is infected with more than one copy of the virus [17]. We, in contrast, chose to use a low inoculation dose of MOI of 0.1. Given the Poisson distribution of HRV infection [18], this MOI should result in about 10% of cells infected with at least one copy of the virus, such as seems to be the case *in vivo*. With this MOI, we utilized two HRV inoculation models.

In the first, continuous, inoculation model, cells were exposed to HRV16 for 24 hours at 37°C (Fig 1A). Then, cell supernatants and lysates were collected for analysis of IFN-β and IL-8 (both: protein and mRNA), while expression of the interferon-responsive gene *OAS1* was quantified in cell lysates (Fig 1A). This model recapitulates the situation in which lower CF airways are constantly exposed to low doses of HRV, such as via droplets generated in the upper airways during the common cold.

In the short-term HRV inoculation model, we exposed cells to HRV16 at MOI of 0.1 for 2 hours at 37°C. This amount of time is sufficient for full absorption of low-titre HRV [19–21]. After that, cell supernatants were aspirated, and cells were washed twice with virus-free culture medium to remove any remaining extracellular virus. Then, the cells were cultured for 22 hours in the virus-free culture medium, following which cell supernatants and lysates were collected to quantify IFN-β, IL-8, and *OAS1* (Fig 1B). This model allows studying the persistence of innate antiviral and inflammatory responses after short-term HRV inoculation.

In addition, the short-term inoculation model was also used to assess the HRV RNA load post-inoculation. After cell inoculation with HRV16 for 2 hours, virus-containing supernatants were removed, cells were washed twice, and cultured for 10, 22 or 34 hours in the virus-free culture medium. At indicated time points, the cells were collected for quantification of the intracellular HRV RNA load (Fig 1C).

Neither continuous nor short-term HRV16 inoculation models were cytotoxic to cells, as verified by MTT assay (data not shown).

### HRV positive and negative strand detection by *in situ* hybridization

For these experiments, we grew cells on coated coverslips and inoculated them with HRV16 as above. At different time points, the cells were washed five times with virus-free culture medium to remove unattached virus and fixed for 30 min in 4% paraformaldehyde. Then, the cells were dehydrated using increasing concentrations of ethanol and stored at -20°C in 100% ethanol pending analyses. For *in situ* hybridization, the cells were rehydrated and processed according to the manufacturer’s protocol using the following probes and dyes: HRV positive strand probe (Affymetrix VF1-10200), HRV negative strand probe (Affymetrix VF1-10170), *ACTB* (β-actin gene to identify the cytoplasm; Affymetrix VA4-10293), and DAPI (to counterstain cell nuclei). The coverslips were mounted onto glass slides using Prolong Diamond fluorescence mounting medium, cured overnight at room temperature, and sealed with a clear nail polish.

The cells were viewed using the LSM780 laser scanning confocal microscope (Zeiss Canada, North York, Canada) of the McGill Cystic Fibrosis Translational Research Centre. The images were obtained using a Plan Apochromat 63X (1.4 NA) immersion oil objective. The *ACTB* probe was excited using 488 nm laser (green signal; emission filter 490–570 nm), whereas both HRV probes were excited using the 561 nm laser (red signal; emission filter 566–697 nm). DAPI was excited using the 405 nm laser (blue signal; emission filter 410–508 nm). Six to eight or four to five z-stack images were obtained from, respectively, primary HBE cells or cell lines. These z-stack images were combined in the ZEN 2010 software (Zeiss Canada) and further processed in Adobe Photoshop (Adobe Creative Suite 5.0; Adobe, San Jose, USA), using only global adjustments of brightness and non-obscuring cropping to enlarge the areas of interest.

### Bacterial virulence products

In some experiments, cells were stimulated with *E*. *coli* LPS (2.5 μg/ml) or *P*. *aeruginosa* flagellin (0.5 μg/ml), or sterile filtrates of *P*. *aeruginosa* culture to compare IL-8 responses to bacterial virulence factors to those stimulated by HRV16. Sterile filtrates were prepared as previously described [22]. Briefly, the culture of late stationary phase *P*. *aeruginosa* (clinical mucoid isolate from a patient with CF) was sterilely filtered to remove bacteria and yield soluble bacterial virulence products. These filtrates were added to cell supernatants at a 1: 100 dilution. This dilution was determined in preliminary experiments not to cause cytotoxicity, as verified by MTT assay (data not shown). In some experiments, the magnitude of stimulation of IL-8 by these bacterial virulence products was compared to that stimulated by the potent pro-inflammatory cytokine IL-1β (10 ng/ml).

In addition, we tested potential adverse effects of *P*. *aeruginosa* filtrates on cell responses to HRV16, as described in Fig 1 and in the Results section.

### Statistical analysis

The data are presented as absolute concentrations of IFN-β or IL-8, or fold increase over basal expression of the above genes and *OAS1* in uninfected cells, or as log_10_ of HRV RNA copy number. The data are presented graphically as box-and-whisker plots to demonstrate median, interquartile range, and min-max values. Statistical analyses were conducted using GraphPad Prism (GraphPad Software; LaJolla, USA) and included descriptive statistics, Mann-Whitney U test, or parametric or non-parametric one-way ANOVA comparisons with, respectively, Tukey or Dunn’s post-hoc tests. The level of statistical confidence was set at p < 0.05.

## Results

### IFN-β, IL-8 and *OAS-1* responses to inoculation with HRV16

**Fig 2:**
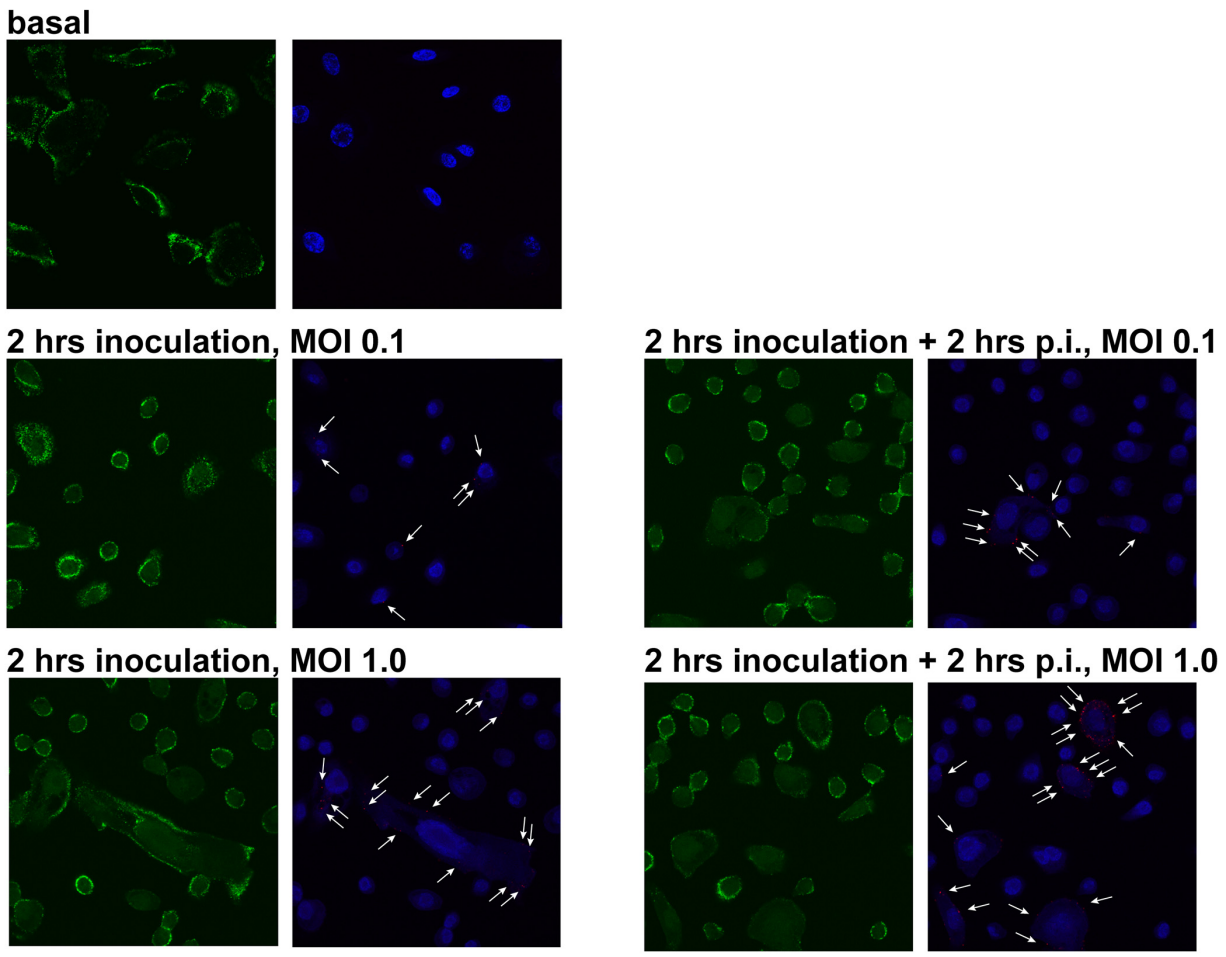
Intracellular HRV RNA load in CF HBE cells after a short-term inoculation with HRV16. Primary CF HBE cells were pre-incubated for 18 hours with experimental culture medium (BEGM without hydrocorticortisone or antibiotics) and subsequently inoculated for 2 hours with HRV16 (HRV) at an MOI of 0.1 or 1.0. Then, virus-containing cell supernatants were removed and cells were rinsed five times with experimental culture medium to deplete extracellular virus. Subsequently, cells presented in left panels were fixed with paraformaldehyde and processed as described in the Materials and Methods to detect the HRV16 positive strand RNA by *in situ* hybridization. Parallel cells (right panels) were incubated for 2 hours post-inoculation (p.i.) and processed similarly to above cells. Green indicates expression of *ACTB* (β-actin gene; used to visualize cell cytoplasm), red dots and white arrows indicate the HRV16 positive strand RNA, and blue is DAPI (nuclear counterstain). The red and blue signals have been slightly overexposed to better visualize HRV16 RNA.

We first wished to compare IFN-β and IL-8 responses in healthy and CF HBE cells continuously inoculated with HRV16 (Fig 1A). As described in the Materials and Methods, we utilized the low inoculation dose of HRV16 (MOI of 0.1). Following the Poisson equation, this inoculation dose was expected to infect a low number of cells, and infected cells should bear low copy numbers of virus RNA. To demonstrate this, we inoculated primary CF HBE cells for 2 hours with HRV16 at an MOI of 0.1. Parallel cells were inoculated with an MOI of 1.0 which is dose most commonly used in the literature. After inoculation, the cells were washed to remove any remaining uninternalized virus, fixed and processed for the *in situ* hybridization assay to detect the HRV16 positive strand. Another set of cells was incubated for additional 2 hours in virus-free culture medium and processed as above. As expected, both the number of infected cells and the average RNA copy number per cell appeared to be higher in the cells inoculated with an MOI of 1.0 (Fig 2). This was true for both time points presented in Fig 2, as well as for earlier time points (data not shown). Therefore, we concluded that inoculation with HRV16 at an MOI of 0.1 leads to a low grade HRV infection, similar to what is documented in clinical specimens, and utilized this MOI in the subsequent experiments.

**Fig 3:**
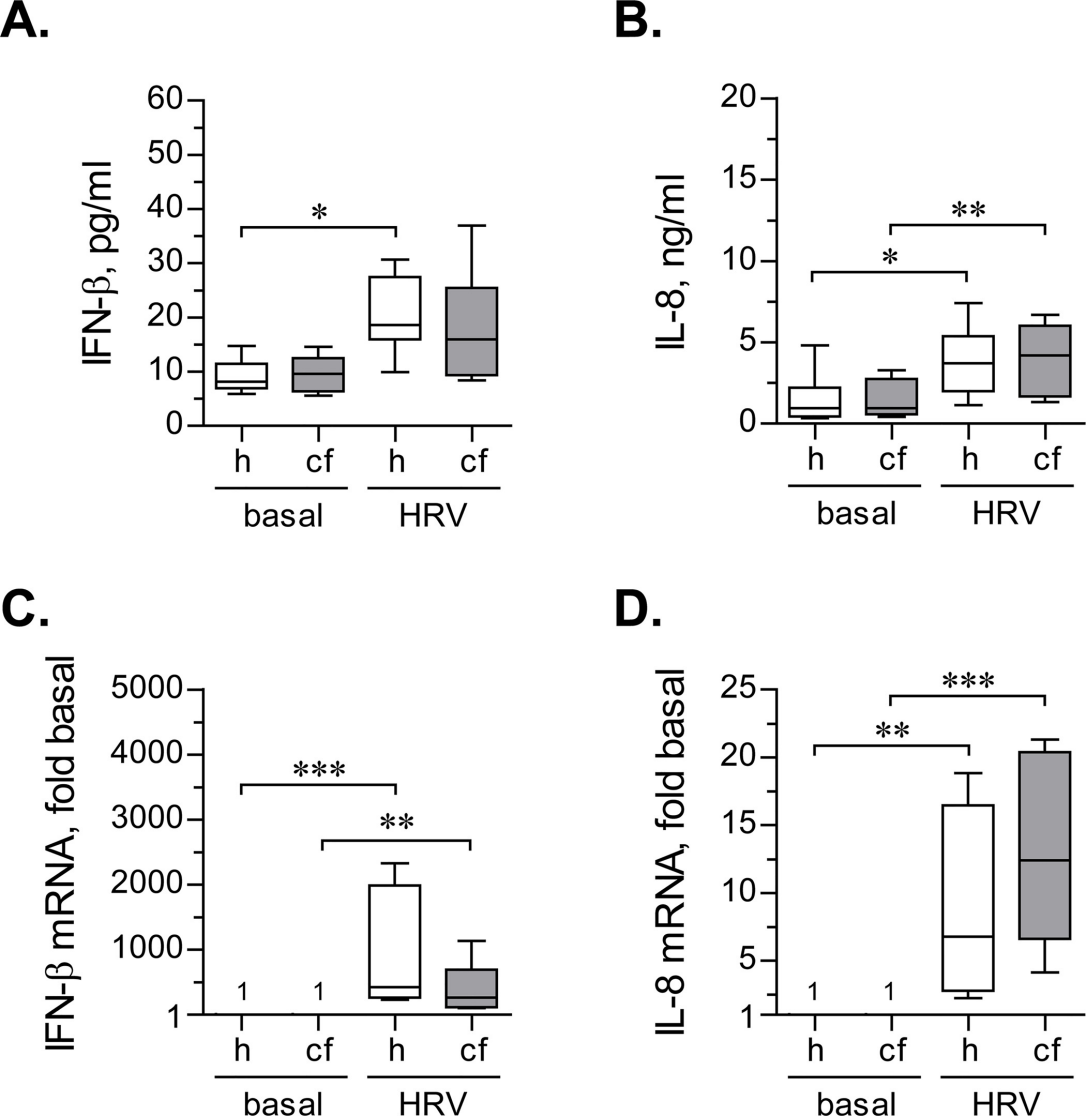
IFN-β and IL-8 response during continuous inoculation with HRV16. (**A**) and (**B**) Primary healthy (h) and CF (cf) HBE cells were pre-incubated for 18 hours with experimental culture medium (BEGM without hydrocorticortisone or antibiotics) and subsequently inoculated for 24 hours with HRV16 (HRV) at an MOI of 0.1. Afterwards, production of IFN-β (**A**) and IL-8 (**B**) was quantified in cell supernatants by respective ELISAs. Data are presented as box-and-whisker plots (medians, interquartile ranges, and min-max values) of absolute values of IFN-β and IL-8 production. n = 8 cultures per group. * p < 0.05 and ** p < 0.01. (**C**) and (**D**) The above cells were lysed, and expressions of mRNA of IFN-β (**C**) and IL-8 (**D**) were quantified by qPCR. Numbers on the plot represent IFN-β mRNA expression in basal cells, assumed as 1. Other data are presented as box-and-whisker plots of fold up-regulation over basal. n = 8 cultures per group. ** p < 0.01 and *** p < 0.001.

Under basal conditions, secretion of IFN-β was comparable between primary healthy and CF HBE cells (Fig 3A). Continuous inoculation with HRV16 caused an approximate two-fold up-regulation of IFN-β secretion in both healthy and CF HBE cells (Fig 3A). This up-regulation became statistically significant only in healthy cells, owing to higher variability of IFN-β production in CF HBE cells (Fig 3A). While IFN-β up-regulation tended to be lower in HRV-infected CF HBE cells (Fig 3A), the differences with healthy HBE cells did not reach statistical significance.

Basal IL-8 secretion was comparable between primary healthy and CF HBE cells (Fig 3B). Both healthy and CF cells mildly, but significantly, up-regulated IL-8 production when infected with HRV16 (Fig 3B). Furthermore, the magnitude of IL-8 up-regulation was comparable between healthy and CF HBE cells.

We next analyzed expression of IFN-β and IL-8 at the mRNA level. IFN-β mRNA was expressed at very low levels under basal conditions. However, continuous inoculation with HRV16 led to a very dramatic (exceeding several hundreds of fold over basal) up-regulation of IFN-β mRNA (Fig 3C). Interestingly, we observed a marked inter-individual variability in the magnitudes of up-regulation of IFN-β mRNA in both healthy and CF HBE cells (Fig 3C). Others also reported a substantial (several log) inter-individual variability of IFN-β mRNA response to HRV [23]. As with secreted protein, the magnitude of IFN-β mRNA up-regulation tended to be lower in CF HBE cells, although the difference with healthy HBE cells did not reach statistical significance (Fig 3C).

Interestingly, in both healthy and CF HBE cells, the magnitude of IFN-β mRNA greatly exceeded production of the secreted protein (hundreds of fold vs. two fold, Fig 3C vs. Fig 3A). The discrepancy between substantial up-regulation of IFN-β mRNA and low amounts of secreted protein was also noted by others [24].

Up-regulation of IL-8 mRNA in HRV-infected CF HBE cells was slightly higher than in healthy cells (Fig 3D), but the observed difference did not reach statistical significance. Furthermore, in contrast to IFN-β, IL-8 showed better agreement between the magnitudes of mRNA vs. secreted protein up-regulation. Specifically, IL-8 mRNA was mildly up-regulated by HRV16 in both healthy and CF HBE cells (<15-fold increase; Fig 3D), and the magnitude of this up-regulation was within the same order of magnitude as that of the secreted protein (respectively, Fig 3D vs. Fig 3B).

Thus, the findings obtained upon continuous inoculation with HRV16 indicated that antiviral and inflammatory responses of CF HBE cells are not significantly different from those in healthy cells. We then tested whether this would also be the case with the short-term HRV16 inoculation model (Fig 1B).

**Fig 4:**
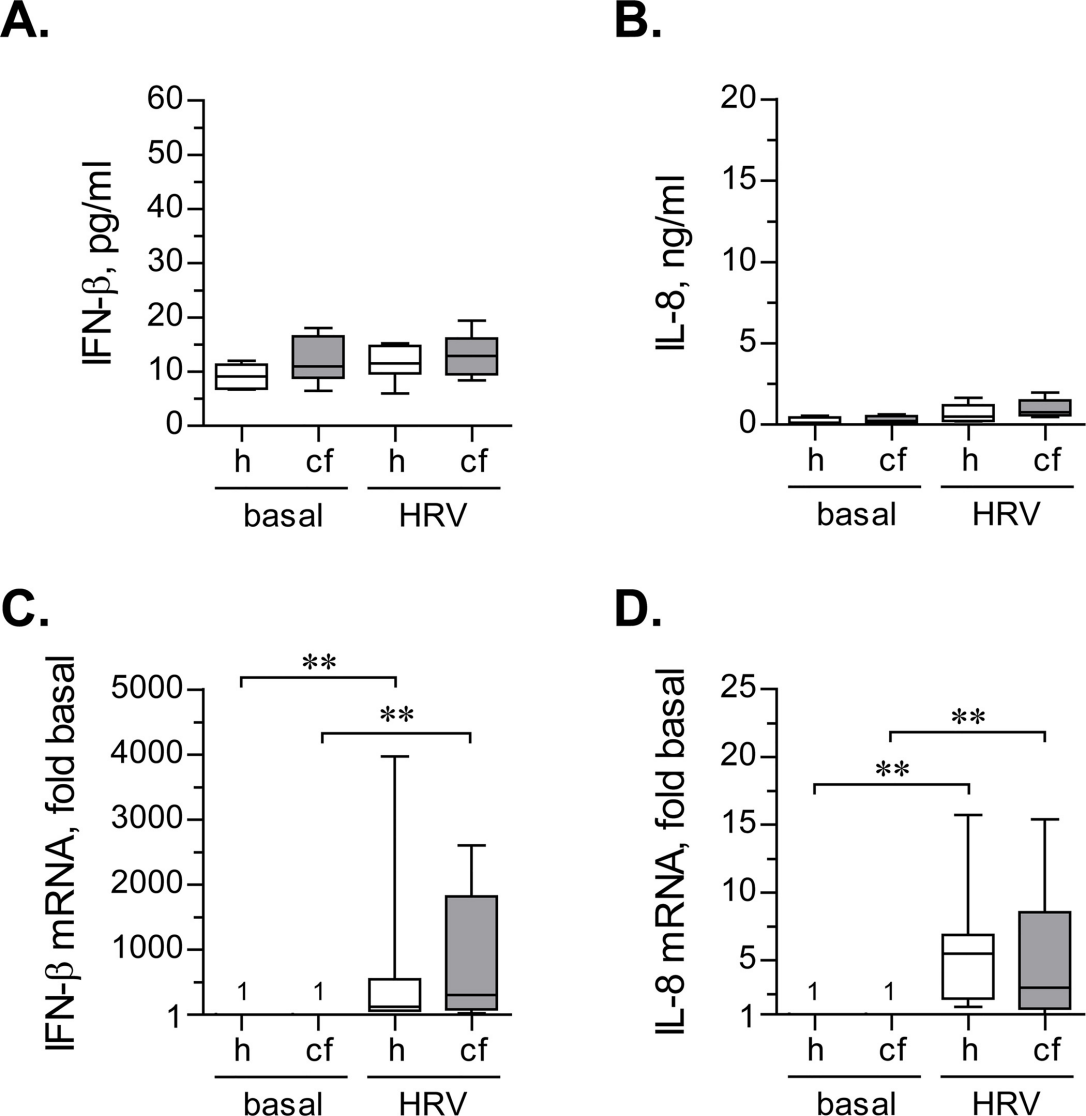
IFN-β and IL-8 response after short-term inoculation with HRV16. (**A**) and (**B**) Primary healthy (h) and CF (cf) HBE cells were pre-incubated for 18 hours with experimental culture medium (BEGM without hydrocorticortisone or antibiotics) and subsequently inoculated for 2 hours with HRV16 (HRV) at an MOI of 0.1. Then, virus-containing cell supernatants were removed, cells were rinsed twice with experimental culture medium to deplete extracellular virus, and incubated for 22 hours without HRV16. Afterwards, production of IFN-β (**A**) and IL-8 (**B**) was quantified in cell supernatants by respective ELISAs. Data are presented as box-and-whisker plots (medians, interquartile ranges, and min-max values) of absolute values of IFN-β and IL-8 production. n = 8 cultures per group. None of the tested differences reached statistical significance. (**C**) and (**D**) The above cells were lysed, and expressions of mRNA of IFN-β (**C**) and IL-8 (**D**) were quantified by qPCR. Numbers on the plot represent IFN-β mRNA expression in basal cells, assumed as 1. Other data are presented as box-and-whisker plots of fold up-regulation over basal. n = 6–7 cultures per group. ** p < 0.01.

Unlike continuous inoculation model, short-term inoculation with HRV16 did not lead to a detectable significant up-regulation of IFN-β secretion (Fig 4A). It should be noted that we utilized high-sensitivity ELISA kit to detect IFN-β, with the lowest level of detection around 1 pg/ml. Using this ELISA kit, we readily detected basal IFN-β secretion in primary HBE cells under all tested conditions. Therefore, the lack of documented up-regulation of secreted IFN-β in HRV-infected cells does not appear to be due to assay limitations. Furthermore, the lack of up-regulation of secreted IFN-β is unlikely to be due to low inoculation dose of HRV16 in our experiments. Others had used HRV at much higher MOIs in a similar short-term inoculation model and also could not detect secreted IFN-β [23].

Similar to IFN-β, IL-8 production was not significantly up-regulated by short-term inoculation with HRV16 (Fig 4B).

We next examined up-regulation of IFN-β and IL-8 mRNAs in primary healthy and CF HBE cells after short-term inoculation with HRV16. In a marked contrast to secreted IFN-β (Fig 4A), we observed a robust up-regulation of its mRNA by HRV16 (Fig 4C), with comparable magnitudes between healthy and CF HBE cells. IL-8 mRNA was also significantly, and to a comparable extent, up-regulated in both healthy and CF HBE cells (Fig 4D).

**Fig 5:**
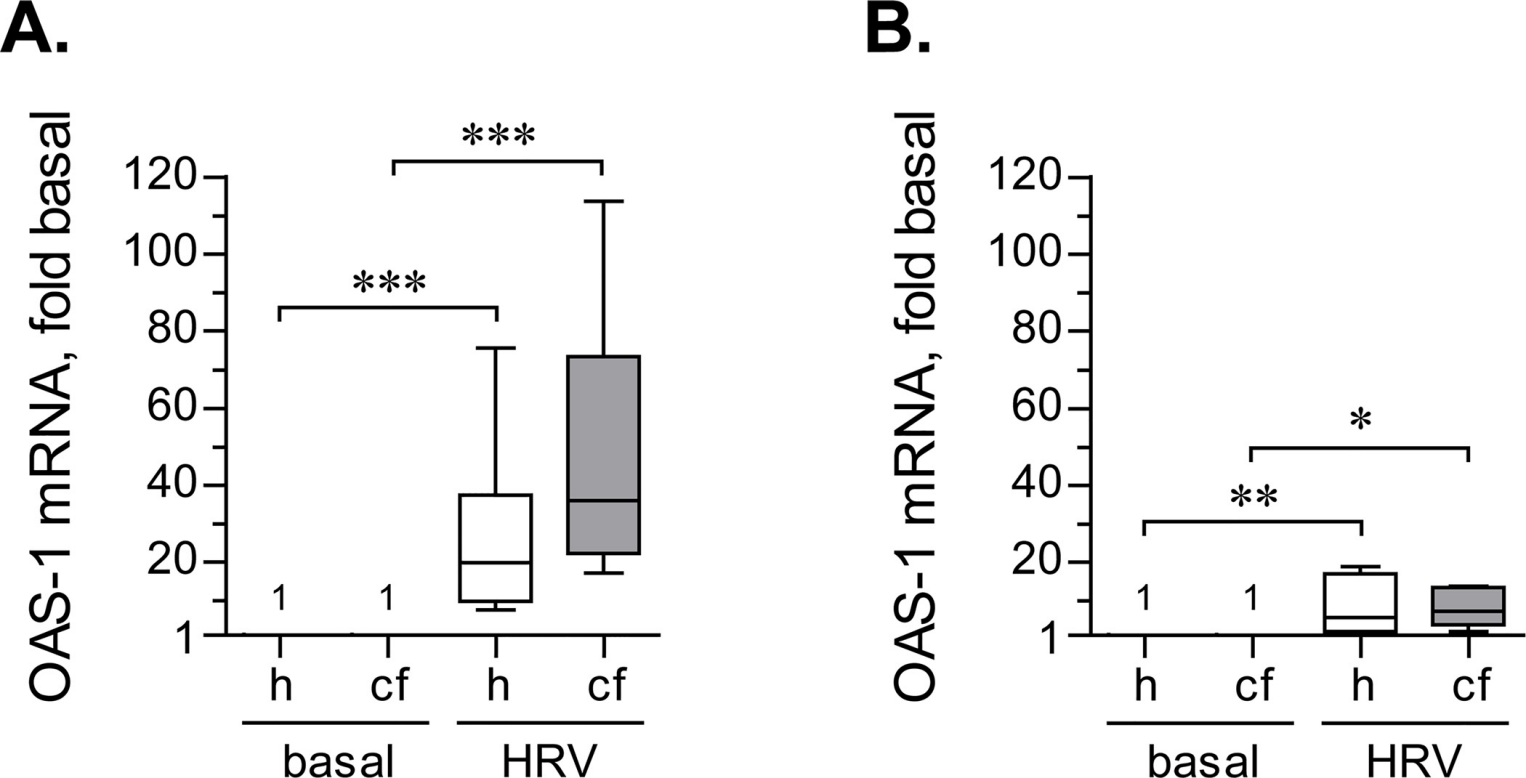
Up-regulation of *OAS1* expression by HRV16. (**A**) Primary healthy (h) and CF (cf) HBE cells were pre-incubated for 18 hours with experimental culture medium (BEGM without hydrocorticortisone or antibiotics) and subsequently inoculated for 24 hours with HRV16 (HRV) at an MOI of 0.1. Afterwards, expression of *OAS1* mRNA was quantified by qPCR. Numbers on the plot represent *OAS1* expression in basal cells, assumed as 1. Other data are presented as box-and-whisker plots (medians, interquartile ranges, and min-max values) of fold up-regulation over basal. n = 8 cultures per group. ** p < 0.01 and *** p < 0.001. (**B**) Primary healthy (h) and CF (cf) HBE cells were pre-incubated as above and subsequently inoculated for 2 hours with HRV16 (HRV) at the MOI of 0.1. Then, virus-containing cell supernatants were removed, cells were rinsed twice with experimental culture medium to deplete extracellular virus, and incubated for 22 hours without HRV16. Afterwards, expression of *OAS1* mRNA was quantified by qPCR. Numbers on the plot represent *OAS1* expression in basal cells, assumed as 1. Other data are presented as box-and-whisker plots of fold up-regulation over basal. n = 6–7 cultures per group. * p < 0.05 and ** p < 0.01.

Both previous experiments demonstrated that when infected with HRV16, CF HBE cells express IFN-β mRNA and secrete the protein at levels not significantly different from healthy HBE cells. We next wished to confirm that CF HBE cells are responsive to secreted IFN-β. To this end, we quantified expression of the interferon-responsive gene *OAS1*. In the continuous HRV16 inoculation model, *OAS1* transcription was up-regulated about 30-fold in healthy HBE cells and even higher (about 50-fold) in CF HBE cells. These up-regulations were significantly different with respect to their respective controls, but not different between CF and healthy HBE cells (Fig 5A).

Short-term inoculation with HRV16 up-regulated *OAS1* to a lesser magnitude (Fig 5B). Specifically, both healthy and CF HBE cells up-regulated *OAS1* by about 10-fold, which was markedly lower than during continuous inoculation (Fig 5B vs. Fig 5A). Again, we observed no significant differences in the magnitudes of *OAS1* up-regulation between HRV-infected healthy and CF HBE cells.

The above experiments did not reveal a significant difference between HRV16-infected primary healthy and CF HBE cells in terms of IFN-β and IL-8 responses, or *OAS-1* up-regulation. However, innate responses of CF airway epithelium may be negatively affected by chronic exposure to virulence factors of opportunistic bacteria colonizing CF airways, such as *P*. *aeruginosa*. Therefore, in the next set of experiments, we assessed potential modulation of these responses to HRV16 by virulence products of *P*. *aeruginosa*.

### Effects of bacterial virulence factors on IFN-β, IL-8, and *OAS-1* in HRV16-infected cells

We first determined which bacterial virulence factors stimulate the strongest innate responses in primary healthy and CF HBE cells. IL-8 was used as a measure of cell innate responses. The cells were stimulated with sterile filtrates of *P*. *aeruginosa* culture, *E*. *coli* LPS, and *P*. *aeruginosa* flagellin. The filtrates mimic epithelial exposure to a mix of diffusible virulence factors of *P*. *aeruginosa* which include pro-oxidants and ligands to Toll-like receptors [25]. With regard to LPS, we utilized *E*. *coli* LPS because *P*. *aeruginosa* LPS did not cause any up-regulation of IL-8 production in primary healthy HBE cells in preliminary experiments (data not shown). *P*. *aeruginosa* flagellin is considered one of the strongest inflammagenic factors of this microorganism [26].

**Fig 6:**
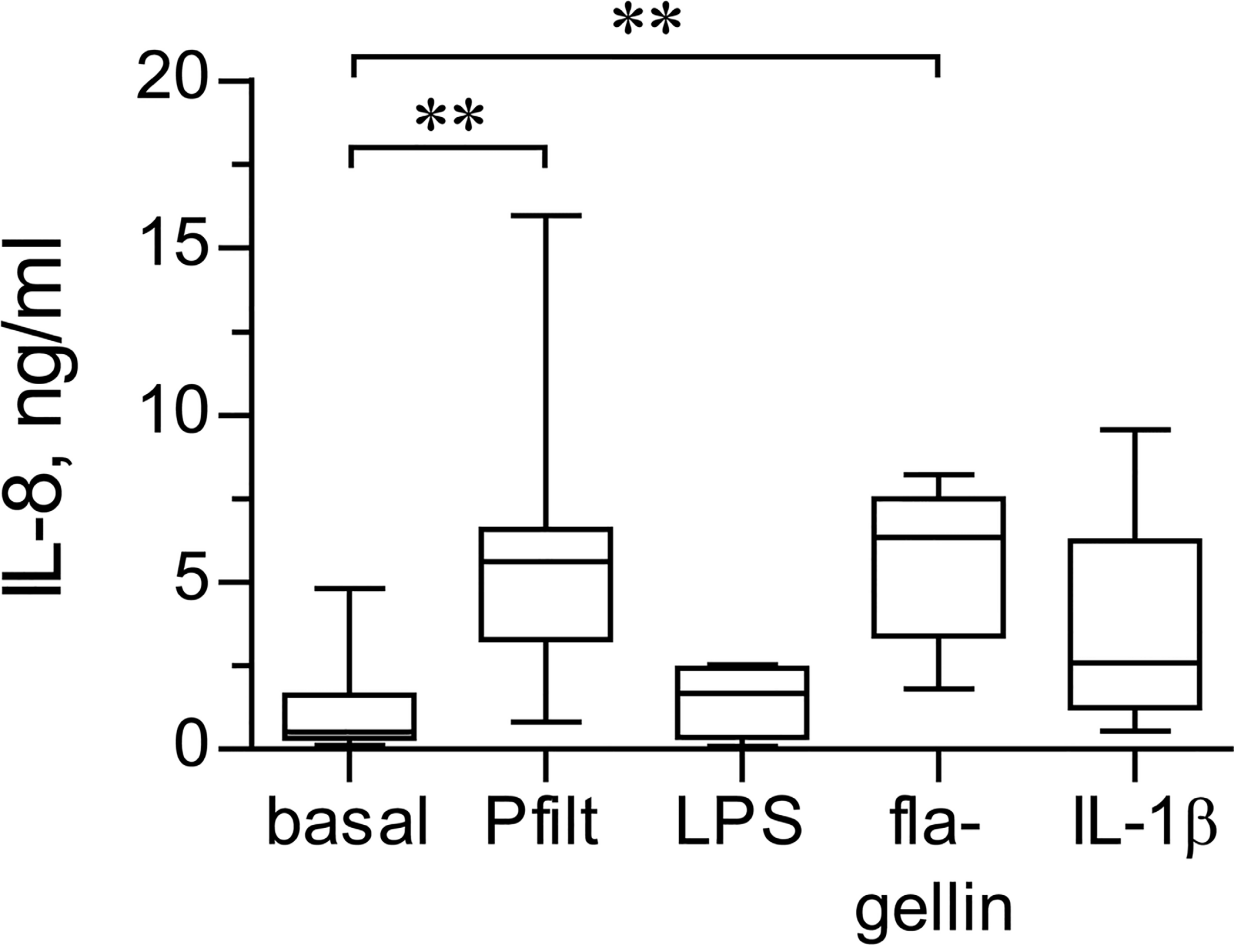
Magnitudes of IL-8 up-regulation by bacterial virulence factors. Primary healthy HBE cells were pre-incubated for 18 hours with experimental culture medium (BEGM without hydrocorticortisone or antibiotics) and subsequently stimulated for 24 hours with sterile filtrates of *P*. *aeriginosa* culture (Pfilt; 1: 100 dilution), *E*. *coli* LPS (2.5 μg/ml), *P*. *aeruginosa* flagellin (0.5 μg/ml), or IL-1β (10 ng/ml). Production of IL-8 was quantified in cell supernatants by ELISA. Data are presented as box-and-whisker plots (medians, interquartile ranges, and min-max values) of absolute values of IL-8 production. n = 5–9 cultures per group. ** p < 0.01.

First, we compared the magnitudes of IL-8 up-regulation by the above bacterial virulence factors. We defined the concentrations of respective stimuli in preliminary experiments, with the criteria being the absence of cytotoxicity and up-regulation of IL-8 by >2-fold over basal. As positive control for IL-8 up-regulation, we stimulated cells with IL-1β, which in our previous studies cause a potent up-regulation of IL-8 in immortalized cell lines [8, 9]. We observed that primary HBE cells up-regulated IL-8 in response to all tested stimuli, but most potently when stimulated with sterile *P*. *aeruginosa* filtrates or flagellin (Fig 6). In fact, IL-8 production stimulated by *P*. *aeruginosa* filtrates and flagellin was up-regulated to a comparable magnitude, and both stimuli caused IL-8 up-regulation exceeding that by IL-1β (Fig 6).

Sterile *P*. *aeruginosa* filtrates contain a mix of bacterial virulence factors [25]; thus, they may mimic more closely the microenvironment of CF airway lumen than e.g., flagellin alone. Therefore, we utilized sterile *P*. *aeruginosa* filtrates in the next experiments to assess the effects of bacterial virulence factors on IFN-β and IL-8 responses in HRV-infected cells. Primary healthy and CF HBE cells were pre-incubated for 18 hours with *P*. *aeruginosa* filtrates and inoculated for 24 hours with HRV16 at an MOI of 0.1 in the presence of these filtrates (Fig 1A).

**Fig 7:**
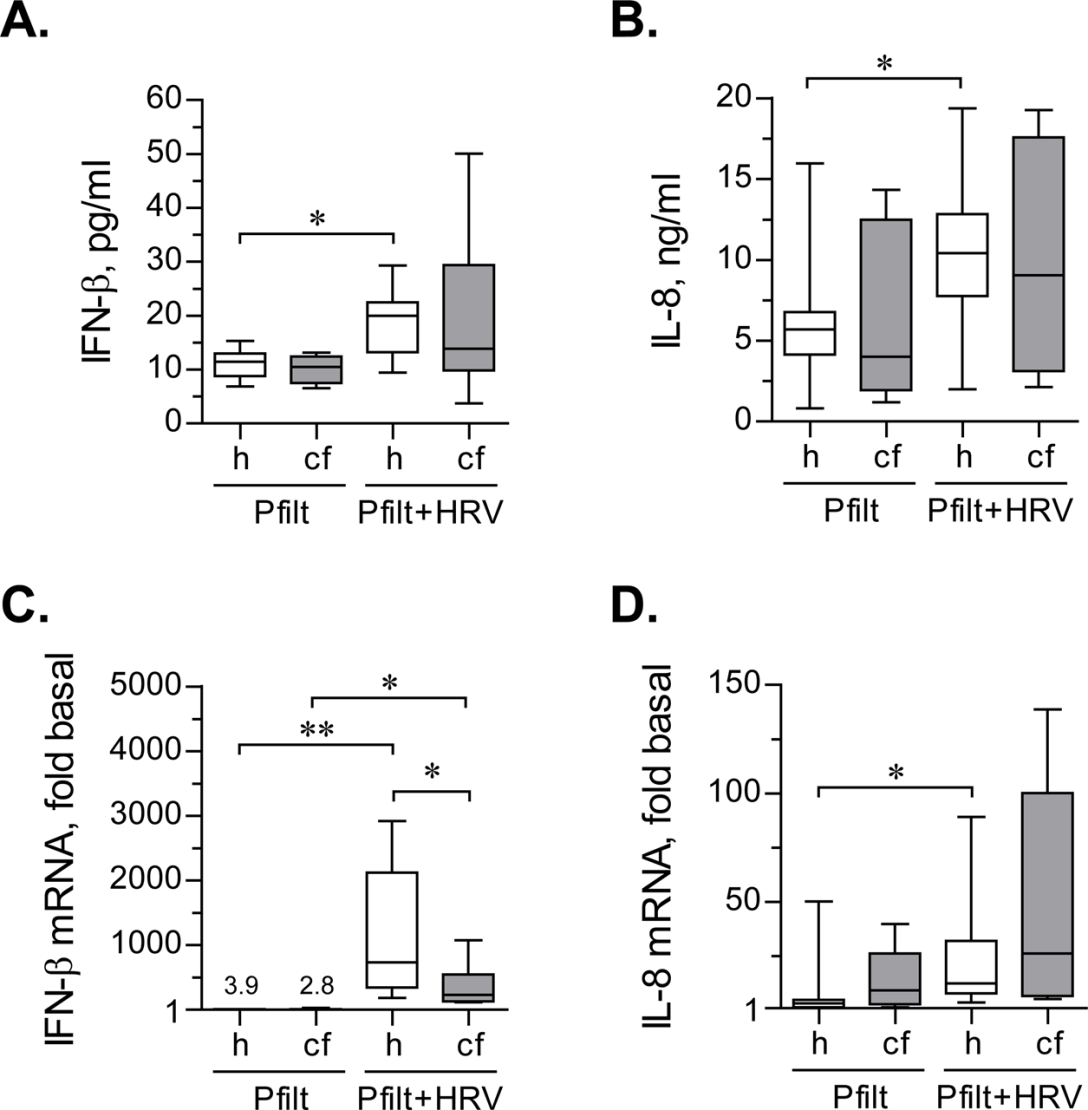
IFN-β and IL-8 response to sterile filtrates of *P*. *aerigunosa* and HRV16. (**A**) and (**B**) Primary healthy (h) and CF (cf) HBE cells were pre-incubated for 18 hours with sterile filtrates of *P*. *aeruginosa* (Pfilt; 1: 100 dilution) in experimental culture medium (BEGM without hydrocorticortisone or antibiotics). Then, cells were inoculated for 24 hours with HRV16 (HRV) at an MOI of 0.1 in the presence of Pfilt. Afterwards, production of IFN-β (**A**) and IL-8 (**B**) was quantified in cell supernatants by respective ELISAs. Data are presented as box-and-whisker plots (medians, interquartile ranges, and min-max values) of absolute values of IFN-β and IL-8 production. n = 8 cultures per group. * p < 0.05. (**C**) and (**D**) The above cells were lysed, and expressions of mRNA of IFN-β (**C**) and IL-8 (**D**) were quantified by qPCR. Expression in basal cells (i.e., without exposure to Pfilt or HRV16; not shown) was assumed as 1. Numbers on the plot represent the medians of IFN-β mRNA up-regulation over basal in Pfilt-stimulated cells; other data are presented as box-and-whisker plots of fold up-regulation over basal. n = 8 cultures per group. * p < 0.05 and ** p < 0.01.

Sterile *P*. *aeruginosa* filtrates did not affect IFN-β production in basal or HRV16-infected healthy HBE cells (Table 1; Fig 7A). However, CF HBE cells showed an insignificant trend toward decreasing IFN-β production (Table 1; Fig 7A).

**Table 1:** IFN-β production stimulated by 24-hour inoculation with HRV16, with or without prior cell exposure to sterile *P*. *aeruginosa* filtrates.

hHBE				cfHBE			
Basal	HRV16	Pfilt	Pfilt+HRV16	Basal	HRV16	Pfilt	Pfilt+HRV16
8.19 (5.92–14.73)	18.65 (9.96–30.66)	11.48 (6.9–15.34)	20.02 (9.47–29.32)	9.58 (5.55–14.58)	15.99 (8.37–36.98)	10.50 (6.53–13.14)	13.88 (3.72–50.07)

Footnote: Data are presented as median (min–max) pg/ml of secreted IFN-β. hHBE: healthy HBE cells; cfHBE: CF HBE cells; Pfilt: sterile filtrates of P. aeruginosa culture. None of the tested differences were significantly different.

When CF HBE cells were stimulated with *P*. *aeruginosa* filtrates, their IL-8 responses were comparable to healthy HBE cells (Table 2; Fig 7B). Therefore, we did not observe the hyperinflammatory phenotype of CF cells, recently documented by us with regard to monocyte-derived macrophages [27]. It is worth noting, though, that CF HBE cells demonstrated somewhat higher variability of IL-8 responses when stimulated with *P*. *aeruginosa* filtrates (Table 2; Fig 7B). We further observed that the filtrates potentiated IL-8 production stimulated by HRV16 infection in both healthy and CF HBE cells (Table 2; Fig 7B), but this potentiation reached significance only with healthy HBE cells.

**Table 2:** IL-8 production stimulated by 24-hour inoculation with HRV16, with or without prior cell exposure to sterile *P*. *aeruginosa* filtrates.

hHBE				cfHBE			
Basal	HRV16	Pfilt	Pfilt+HRV16	Basal	HRV16	Pfilt	Pfilt+HRV16
0.97 (0.34–4.81)	3.71 (4.47–7.41)	5.69* (0.81–15.97)	10.43^#^ (2.01–19.38)	0.96 (0.42–3.29)	4.19 (1.33–6.68)	4.01* (1.18–14.34)	9.04 (2.12–19.25)

Footnote: Data are presented as median (min–max) ng/ml of secreted IL-8. hHBE: healthy HBE cells; cfHBE: CF HBE cells; Pfilt: sterile filtrates of P. aeruginosa culture.

* *p < 0*.*05 vs*. *basal*

^*#*^ *p < 0*.*01 vs*. *HRV16*

Upon quantifying IFN-β and IL-8 mRNA expression, we observed that sterile *P*. *aeruginosa* filtrates mildly up-regulated IFN-β mRNA expression (Fig 7C). This could be due to signalling through Toll-like Receptor 4, which can mildly stimulate IFN-β transcription [28]. Furthermore, IFN-β mRNA expression in HRV16-infected healthy HBE cells and exposed to sterile *P*. *aeruginosa* filtrates was significantly higher than in their CF counterparts (Fig 7C).

As expected, sterile *P*. *aeruginosa* filtrates up-regulated IL-8 mRNA (Fig 7D). In addition, the filtrates significantly potentiated IL-8 mRNA up-regulation in healthy HBE cells infected with HRV16 (Fig 7D). CF cells exhibited a similar trend, albeit without reaching statistical significance (Fig 7D).

**Fig 8:**
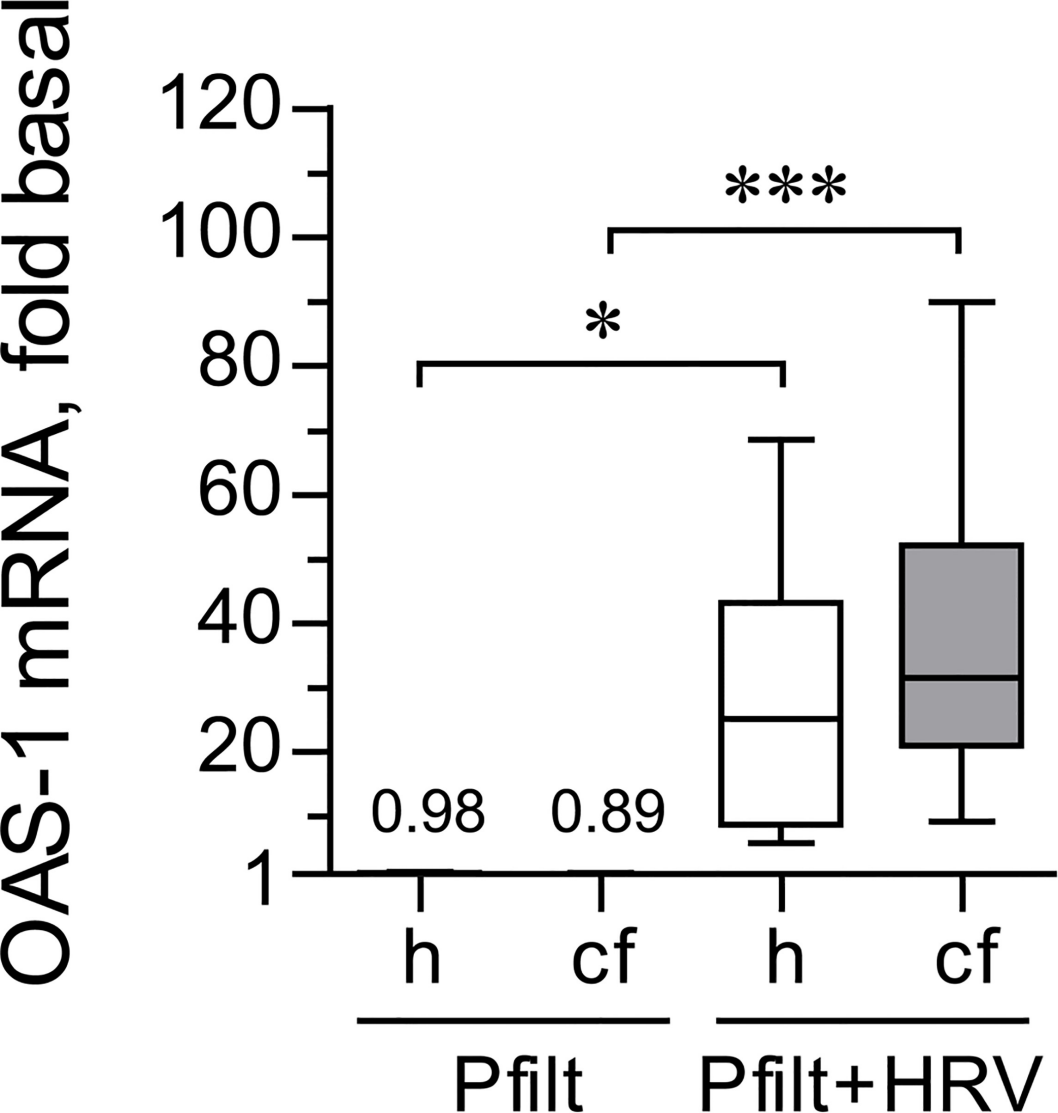
*OAS1* response to sterile filtrates of *P*. *aerigunosa* and HRV16. Primary healthy (h) and CF (cf) HBE cells were pre-incubated for 18 hours with sterile filtrates of *P*. *aeruginosa* (Pfilt; 1: 100 dilution) in experimental culture medium (BEGM without hydrocorticortisone or antibiotics). Then, cells were inoculated for 24 hours with HRV16 (HRV) at an MOI of 0.1 in the presence of Pfilt. Afterwards, expression of *OAS1* mRNA was quantified by qPCR. Expression in basal cells (i.e., without exposure to Pfilt or HRV16; not shown) was assumed as 1. Numbers on the plot represent the medians of IFN-β mRNA up-regulation over basal in Pfilt-stimulated cells; other data are presented as box-and-whisker plots of fold up-regulation over basal. n = 8 cultures per group. * p < 0.05 and *** p < 0.001.

We finally tested whether prior exposure to sterile *P*. *aeruginosa* filtrates would affect expression of *OAS1* during HRV16 infection. We observed that *OAS1* expression was potently up-regulated by HRV16 even in the presence of the filtrates (Fig 8). Furthermore, the magnitude of up-regulation of *OAS1* expression by HRV16 was not affected by the filtrates in either healthy or CF HBE cells (Fig 8 vs. Fig 5A; p > 0.5 for all comparisons).

These experiments demonstrated that IL-8 production stimulated by low-grade HRV16 infection is further potentiated in the presence of virulence factors of *P*. *aeruginosa*, the most common opportunistic pathogen colonizing CF airways. In contrast, secreted IFN-β and, more so, IFN-β mRNA may be suppressed by virulence factors of *P*. *aeruginosa* in CF HBE cells. However, the response of CF HBE cells to IFN-β (i.e., *OAS1* expression) did not appear to be affected by virulence factors of *P*. *aeruginosa*.

We then tested whether intracellular HRV16 RNA load would be different between healthy and CF HBE cells. Since we observed some negative modulation of the IFN-β response by sterile filtrates of *P*. *aeruginosa*, we also tested whether HRV16 RNA load would be negatively affected by these filtrates.

### HRV16 RNA load in healthy and CF HBE cells

In the following experiments, we chose to use the short-term inoculation with HRV16 to allow for the assessment of kinetics of HRV16 RNA load, with or without prior exposure to sterile filtrates of *P*. *aeruginosa* (Fig 1C).

**Fig 9:**
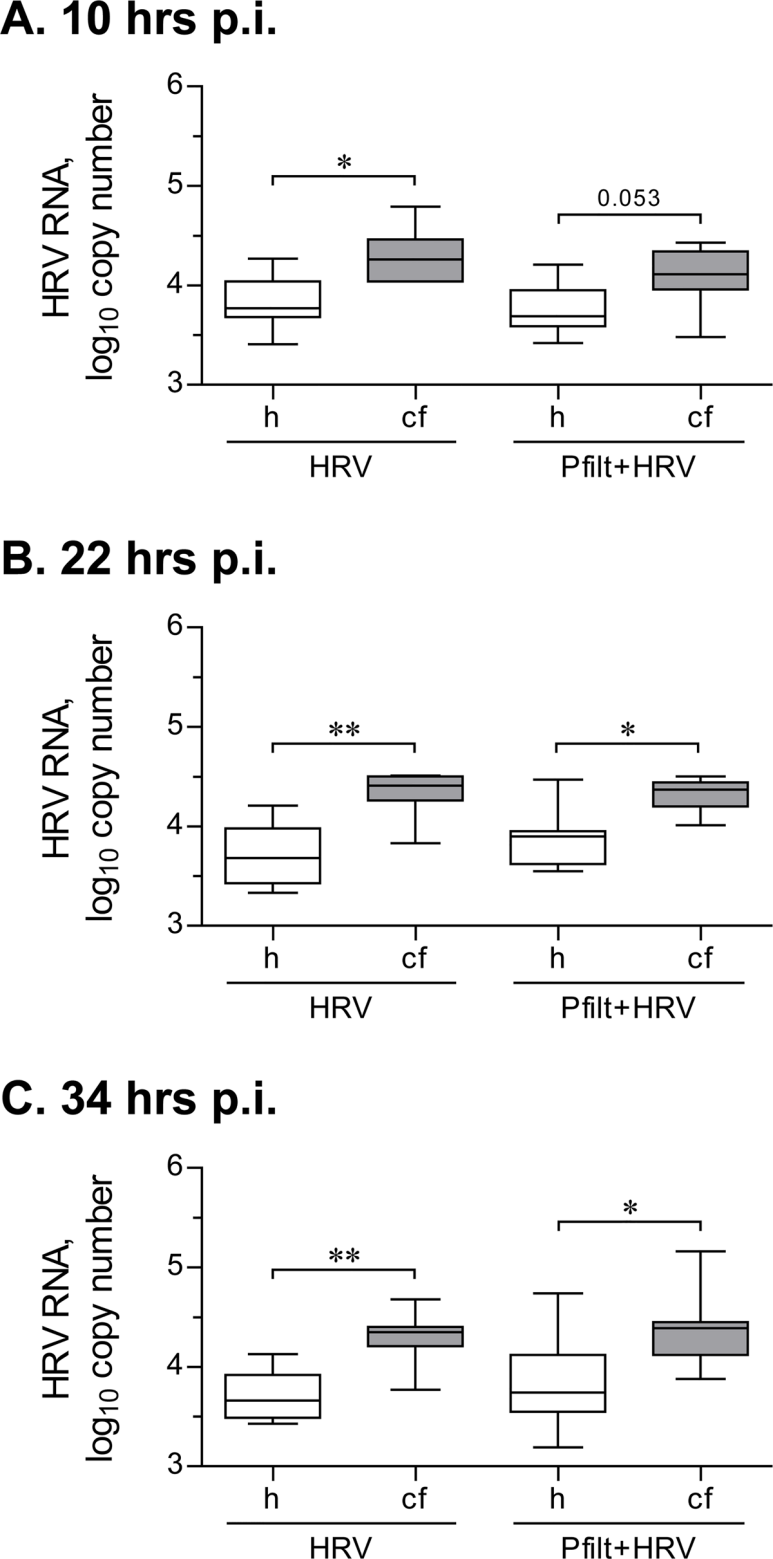
Intracellular HRV RNA load after a short-term inoculation with HRV16. Primary healthy (h) and CF (cf) HBE were pre-incubated for 18 hours with experimental culture medium (BEGM without hydrocorticortisone or antibiotics), with or without sterile filtrates of *P*. *aeruginosa* culture (Pfilt; 1: 100 dilution), and were subsequently inoculated for 2 hours with HRV16 (HRV) at an MOI of 0.1. Then, virus-containing cell supernatants were removed, and cells were rinsed twice with experimental culture medium to deplete extracellular virus. Afterwards, cells were incubated without HRV, with or without Pfilt, and collected at 10 hours (**A**), 22 hours (**B**), and 34 hours (**C**) post-inoculation (p.i.). HRV RNA copy numbers were quantified by qPCR against a serially diluted HRV16 standard with known copy numbers. Data are presented as box-and-whisker plots (medians, interquartile ranges, and min-max values) of log_10_ HRV copy numbers. n = 8 cultures per group. * p < 0.05 and ** p < 0.01.

We observed that HVR16 RNA load was significantly higher in CF HBE cells at 10 hours post-inoculation (Fig 9A). Interestingly, the median copy number of intracellular HRV16 RNA did not change significantly during the later time points (i.e., 22 and 34 hours post-inoculation) in both healthy and CF HBE cells (Fig 9B and 9C vs. Fig 9A). This could potentially indicate that virus replication had stabilized at the later time points. Previous studies on HRV replication kinetics have also documented similar stabilization of virus replication [29, 30]. This replication “plateau” is reached faster when lower inoculation doses of HRV are utilized [29], and this seems to be the case in our model. We then attempted to document HRV replication by performing *in situ* hybridization of the HRV16 negative strand, which is produced during intracellular virus replication. However, we could not detect the negative strand. Others, using qPCR, demonstrated that the abundance of the negative strand is almost two orders of magnitude lower than that of the positive strand [31]. Given the low abundance of virus copies in our experimental model, the lack of detection of the negative strand was likely because it was beyond the limits of detection in our low inoculation model.

Importantly, over the three studied time points, we observed that CF HBE cells demonstrated significantly higher copy numbers of HRV16 RNA (Fig 9A–9C). Here, our findings are in a good agreement with recent studies reporting elevated HRV RNA copy numbers in CF HBE cells 24–48 hours post-inoculation, even though these studies utilized HRV1b (a minor group strain) and a much higher inoculation dose (MOI of 4 or 25) [23, 32]. Furthermore, in previous reports, elevated intracellular HRV RNA copy numbers were corroborated by higher infectivity of corresponding cell culture supernatants [23], confirming the validity of RNA quantification as a measure of virus load.

Pre-exposure to sterile filtrates of *P*. *aeruginosa* did not alter HRV16 RNA copy numbers in healthy or CF cells at any of the studied time points (Fig 9A–9C). This was consistent with our preceding findings that indicated a preserved *OAS1* up-regulation in CF HBE cells infected with HRV16 after pre-exposure to sterile filtrates of *P*. *aeruginosa* (Fig 8).

Since high HRV RNA loads were observed by us and others [23] in the absence of pre-exposure to bacterial virulence factors, we next asked whether this could be due to mechanisms intrinsic to the CF gene mutation. We, therefore, sought to confirm that the observed differences in HRV16 RNA load between CF and non-CF cells would be reproducible in cells bearing the delF508 *CFTR* mutation. In this regard, we tested isogenic cell lines CFBE41o- dF and WT which respectively overexpress delF508 or wild-type *CFTR*. To control for potential artifacts of *CFTR* overexpression, we also tested parental CFBE41o- which expresses endogenous levels of delF508 *CFTR*.

**Fig 10:**
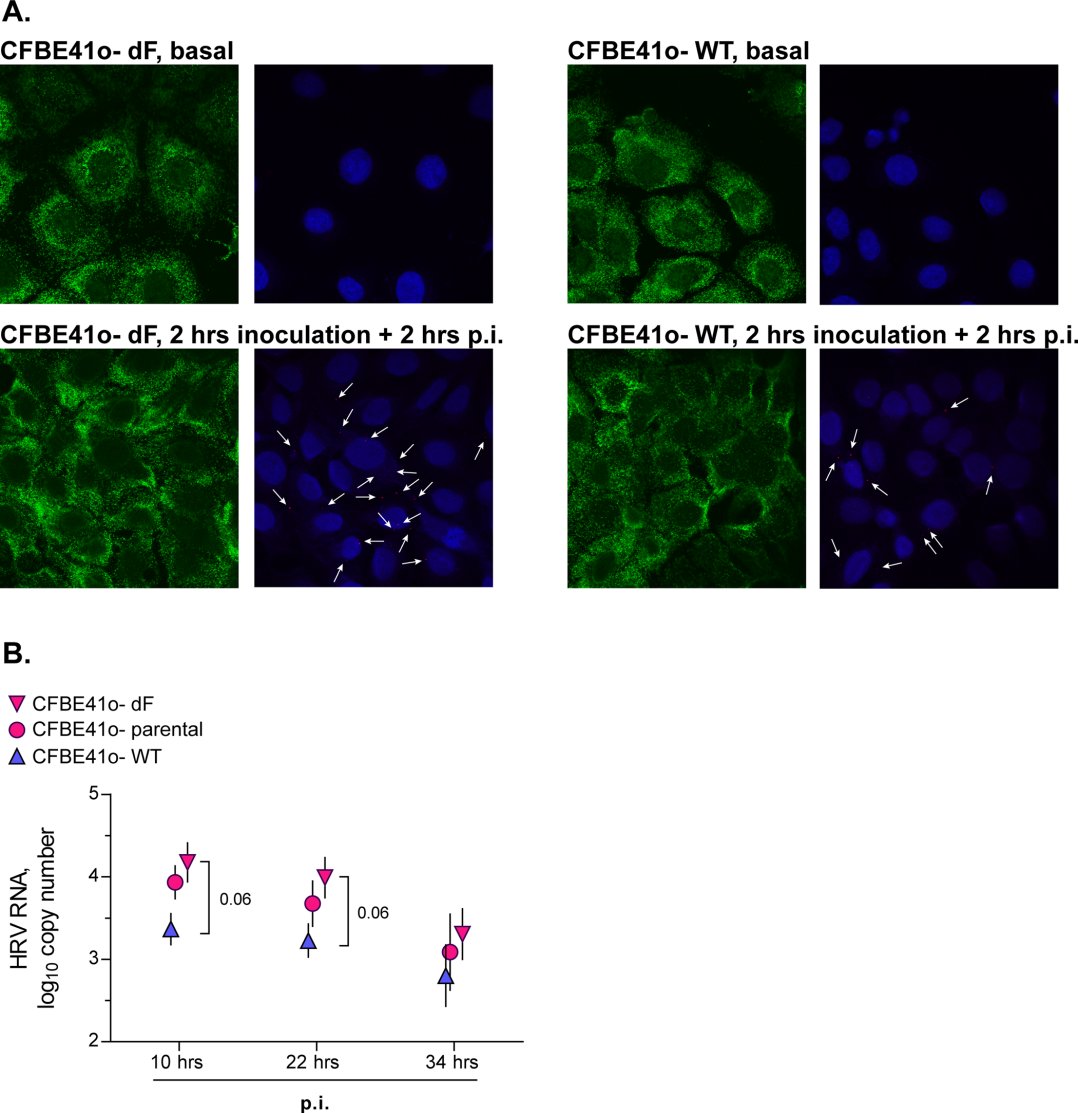
Intracellular HRV RNA load in cell lines expressing delF508 or wild-type *CFTR* after a short-term inoculation with HRV16. (**A**) Isogenic bronchial epithelial cell lines CFBE41o- dF and WT that, respectively, overexpress delF508 or wild-type *CFTR* were inoculated for 2 hours with HRV16 (HRV) at an MOI of 0.1. Then, virus-containing cell supernatants were removed, cells were rinsed twice with culture medium to deplete extracellular virus, and were incubated for 2 hours post-inoculation (p.i.) HRV. Then, cells were fixed with paraformaldehyde and processed as described in the Materials and Methods to detect the HRV16 positive strand RNA by *in situ* hybridization. Green indicates expression of *ACTB* (β-actin gene; used to visualize cell cytoplasm), red dots and white arrows indicate the HRV16 positive strand RNA, and blue is DAPI (nuclear counterstain). The red and blue signals have been slightly overexposed to better visualize HRV16 RNA. (**B**) The above isogenic cell lines and parental cell line CFBE41o- were inoculated for 2 hours with HRV16 (HRV) at an MOI of 0.1. Then, virus-containing cell supernatants were removed, cells were rinsed twice with culture medium to deplete extracellular virus, and incubated for 10, 22, and 34 hours post-inoculation (p.i.). HRV RNA copy numbers were quantified by qPCR against a serially diluted HRV16 standard with known copy numbers. Data are presented as mean ± SEM of log_10_ HRV copy numbers of n = 4–5 independent experiments.

First, we visualized the HRV16 positive strand in isogenic cell lines by *in situ* hybridization. We observed that CFBE41o- dF cells appeared to harbour higher numbers of HRV16 RNA copies (Fig 10A). We next wished to confirm this observation by quantifying HRV16 RNA copy numbers by qPCR. Confirming the preceding observations, both parental CFBE41o- and CFBE41o- dF cell lines yielded more copies of HRV16 RNA (Fig 10B), especially during the early hours post-inoculation with the virus. This confirms that cells expressing delF508 *CFTR* are more prone to contain higher intracellular virus RNA load.

Interestingly, in the tested cell lines, viral RNA copy numbers markedly decreased over the observed period of time (Fig 10B), which was in contrast to observations in primary HBE cells (Fig 9). As a result, the differences in HRV16 RNA loads observed at 10 and 22 hours post-inoculation were diminished at 34 hours post-inoculation (Fig 10B). This observation, along with the results of the *in situ* hybridization assay (Fig 10A), further underscores that high HRV RNA load in CF HBE cells is likely to occur due to early events that take place either during or post-inoculation with the virus.

## Discussion

Patients with CF often develop lower airway exacerbations following HRV infections in the upper respiratory tract. The pathogenesis of these exacerbations is not clear, which prevents developing targeted therapies. Several studies with primary HBE cells have been conducted to date, but the results do not fully clarify the phenomena observed in patients, such as high virus load and heightened IL-8 levels in lower airway secretions [5]. This prompted us to conduct the above studies to better elucidate the vulnerability of CF cells to HRV infections.

In the present study, we demonstrate that, overall, primary CF HBE cells infected with HRV16 secrete the antiviral cytokine IFN-β and up-regulate the interferon-responsive gene *OAS1* to magnitudes not significantly different from those in healthy cells. Yet, intracellular virus RNA load is higher in CF HBE cells, as shown by us and others [23, 32]. Furthermore, we and others demonstrate that high intracellular virus load may persist in CF cells despite the seemingly functional IFN-β response. Importantly, this phenomenon is not limited to a particular HRV strain or inoculation dose. Firstly, the strains of HRV utilized in our and previous studies [23, 32] bind to different extracellular receptors. Secondly, ours and previous studies covered a wide range of inoculation doses (from the MOI of 0.1 in our study to up to the MOI of 25 in [23, 32]). Importantly, high intracellular HRV load occurs in CF HBE cells even in the absence of bacteria or bacterial virulence factors. Therefore, these defects appear to be intrinsic to CF cells. Our observations with CF isogenic cell lines provide further supporting evidence for the intrinsic character of the defects in cells expressing delF508 *CFTR*.

Our experimental observations give rise to two follow-up questions. First, how can HRV load be elevated in CF HBE cells, if IFN-β expression, production, and downstream effects on CF cells–gauged by the expression of *OAS1* –are comparable to those in healthy cells? Second: if the IFN-β response is not deficient, what could cause the elevated HRV load in CF cells?

The first question can potentially be answered by further studies focusing on antiviral responses that occur during the early hours of HRV infection. We demonstrate in this report that elevated HRV RNA load can be detected in as early as 2 hours post-inoculation with the virus. Early intracellular HRV load may be determined by IFNs that are expressed before IFN-β. Specifically, IFN-α or -λ are expressed prior to up-regulation of IFN-β mRNA [33]. It is possible that IFN-β may not define the early virus load to the same extent as e.g., IFN-α or -λ. Since IFN-α and -λ, similar to IFN-β, exert their antiviral effects via increased transcription of interferon-responsive genes, future studies should assess early expression of IFNs and interferon-responsive genes. In this regard, we assessed here the expression of one such gene, *OAS1*, and found this expression to be comparable between HRV-infected healthy and CF HBE cells. However, a more comprehensive assessment may be needed to verify that HRV-infected CF HBE cells up-regulate a panel of interferon-responsive genes both qualitatively and quantitatively similar to healthy HBE cells. Furthermore, our IFN-β and *OAS-1* quantification was done after 24 hours of infection, whereas–given our present findings–it is interesting to assess the expression of interferon-responsive genes in the time frame of 0–10 hours post-inoculation with the virus.

The second question–what causes high HRV RNA load in CF HBE cells–may also require in-depth studies of early events of HRV infection. One could speculate that the differences between healthy and CF cells may occur at the step of HRV internalization. Virus binding to cell receptors is less likely to be the culprit, because high intracellular HRV load was documented using HRV16 (our study) and HRV1b [23]. These HRV subtypes use different receptors (respectively, ICAM-1 [34, 35] and members of the LDL receptor group [36]). This excludes abnormalities at the level of virus binding to the receptor. Since HRV, regardless of the strain, is internalized via endosomal uptake, this step may potentially be the one at fault in CF. Indeed, if HRV uptake into and processing by the CF endosomes is altered, such as was shown for Toll-like Receptor 4 in CF cells [37, 38], this may result in a different kinetics of virus internalization in CF cells, pre-disposing them to higher intracellular virus loads.

CF primary cultures and CF cell lines utilized in our study originated from patients with the type of *CFTR* mutation that causes misfolding of CFTR protein in the endoplasmic reticulum, endoplasmatic reticulum stress, and the unfolded protein response. This occurs in addition to deficient transmembrane electrolyte transport, since CFTR protein serves as an electrolyte channel. At this stage, one could only speculate which of these abnormal processes cause elevated HRV load. But if *CFTR* mutation predisposes to increased HRV internalization, it is interesting to examine whether a *CFTR* corrector would rectify this deficiency. There are approved correctors currently being tested in clinical studies [39] which protect mutant protein from misfolding and premature degradation, and restore to a certain degree its electrolyte function. If HRV internalization is increased in CF airway epithelium due to CFTR protein misfolding or deficient electrolyte function, then such pharmacological corrector may help diminish intracellular virus load. If the association between mutant *CFTR* and increased intracellular HRV load is due to other factors, then IFN-augmenting strategies may be of help until the underlying mechanisms are uncovered and appropriate molecular therapies designed.

If high HRV load in CF cells arises from mechanisms upstream of innate antiviral responses, then one may ask whether IFN-β augmentation would help facilitate virus clearance in CF HBE cells, given that this cytokine does not appear to be deficient in CF. IFN-β can be augmented by exogenous supplementation [40] or by stimulating virus-sensing receptors [41]. We believe that IFN-β augmentation would still be beneficial in CF. Studies demonstrate that IFN-β augmentation facilitates virus clearance in HRV-infected healthy HBE cells [42], whose IFN-β responses are, by definition, “normal”. Indeed, the value of IFN-β augmentation in CF requires further studies.

It is worth mentioning that we and others [24], respectively using diffusible virulence factors of *P*. *aeruginosa* or live pathogen, demonstrate vulnerability of CF HBE cells to immunosuppressing effects of this pathogen. In addition, we observed that virulence factors of *P*. *aeruginosa* potentiate IL-8 production in HRV-infected CF HBE cells. It is, therefore, possible that chronic inflammation in CF airways colonized with *P*. *aeruginosa* is aggravated by HRV through the suppression of the antiviral and potentiation of the inflammatory responses.

A limitation of our study is that experiments were conducted in submerged cell culture. Whereas the majority of HBE cells are present in a well-differentiated state in healthy airways, there is likely to be some degree of turnover and replacement by proliferating cells, and submerged HBE cells may be seen as a model for proliferating cells. The relative proportion of these proliferating cells may increase in diseased conditions. Well-differentiated HBE cells, mimicked by primary cells’ growth at the air-liquid interface, were believed to be poorly infectable by HRV [43]. A particular susceptibility of patients with chronic inflammatory lung disease, such as CF, to HRV-associated exacerbations was often discussed in the context of prior epithelial damage and the presence of poorly differentiated cells [43], which were thought to be the target of HRV. Also, the great majority of the studies with HRV and CF cells conducted to date were done in submerged cells, and we wanted to compare our findings against those by others, thus conducting the present experiments with submerged cells. In addition, well-differentiated cell culture requires seeding the cells on semi-permeable supports at high initial concentrations. Therefore, studies with well-differentiated cells are inherently limited in the number of experiments that can be conducted in parallel with the same culture. Since we wanted to test multiple outcomes under two inoculating conditions, we resorted to studying cells under submerged conditions.

A very recent report [44], which was published after our study had been concluded, demonstrated that well-differentiated cells can be infected by HRV if continuously inoculated with the virus. Interestingly, our study also demonstrates that continuous inoculation with the virus leads to a different cell response pattern. For example, we documented up-regulation of secreted IFN-β in HRV16-infected HBE cells, a phenomenon rarely observed in prior studies. With our study drawing attention to early events of HRV infection and with demonstrated feasibility to infect well-differentiated HBE cells [44], future studies should incorporate experiments with cells cultured at the air-liquid interface.

## Conclusions

Our study demonstrates increased intracellular HRV RNA load in CF HBE cells, which occurs during the early hours of virus infection despite preserved IFN-β and *OAS1* responses. Further studies on virus internalization, endosomal processing, and early antiviral responses in CF HBE cells, including well-differentiated cells, are needed to define the *CFTR*-related abnormalities and develop targeted antiviral therapies.
